# Incidence of and predisposing factors for pseudoaneurysm formation in a high-volume cardiovascular center

**DOI:** 10.1371/journal.pone.0256317

**Published:** 2021-08-24

**Authors:** Hunor Sarkadi, Judit Csőre, Dániel Sándor Veres, Nándor Szegedi, Levente Molnár, László Gellér, Viktor Bérczi, Edit Dósa

**Affiliations:** 1 Heart and Vascular Center, Semmelweis University, Budapest, Hungary; 2 Department of Biophysics and Radiation Biology, Semmelweis University, Budapest, Hungary; 3 Medical Imaging Center, Semmelweis University, Budapest, Hungary; 4 Hungarian Vascular Radiology Research Group, Semmelweis University, Budapest, Hungary; University of Bern, University Hospital Bern, SWITZERLAND

## Abstract

**Purpose:**

To evaluate factors associated with pseudoaneurysm (PSA) development.

**Methods:**

Between January 2016 and May 2020, 30,196 patients had invasive vascular radiological or cardiac endovascular procedures that required arterial puncture. All patients with PSA were identified. A matched (age, gender, and type of the procedure) control group of 134 patients was created to reveal predictors of PSA formation.

**Results:**

Single PSAs were found in 134 patients. Fifty-three PSAs developed after radiological procedures (53/6555 [0.8%]), 31 after coronary artery procedures (31/18038 [0.2%]), 25 after non-coronary artery cardiac procedures (25/5603 [0.4%]), and 25 due to procedures in which the arterial puncture was unintended. Thirty-four PSAs (25.4%) were localized to the upper extremity arteries (vascular closure device [VCD], N = 0), while 100 (74.6%) arose from the lower extremity arteries (VCD, N = 37). The PSA prevalence was 0.05% (10/20478) in the radial artery, 0.1% (2/1818) in the ulnar artery, 1.2% (22/1897) in the brachial artery, and 0.4% (99/22202) in the femoral artery. Treatments for upper and lower limb PSAs were as follows: bandage replacement (32.4% and 14%, respectively), ultrasound-guided compression (11.8% and 1%, respectively), ultrasound-guided thrombin injection (38.2% and 78%, respectively), and open surgery (17.6% and 12%, respectively). Reintervention was necessary in 19 patients (14.2%). The prevalence of PSA for the punctured artery with and without VCD use was 37/3555 (1%) and 97/27204 (0.4%), respectively (OR, 2.94; 95% CI, 1.95–4.34; P<0.001). The effect of red blood cell (RBC) count (P<0.001), hematocrit value (P<0.001), hemoglobin value (P<0.001), international normalized ratio (INR; P<0.001), RBC count—INR interaction (P = 0.003), and RBC count—VCD use interaction (P = 0.036) on PSA formation was significant.

**Conclusion:**

Patients in whom the puncture site is closed with a VCD require increased observation. Preprocedural laboratory findings are useful for the identification of patients at high risk of PSA formation.

## Introduction

Minimally invasive endovascular procedures are becoming increasingly popular worldwide. However, they can be associated with systemic (e.g., contrast-induced reactions such as allergy or nephropathy), as well as treatment-related local complications [1–3]. Pseudoaneurysm (PSA) development is the second most common complication in those undergoing procedures that require an arterial puncture [4]. Its prevalence has been reported to range from 0.05% to 6%, with a higher incidence after therapeutic interventions than after diagnostic catheterizations [1, 4–7]. The etiology of PSA is multifactorial. The site of the puncture, size of the sheath inserted, complexity and length of the procedure, and anticoagulant therapy applied, among others, are known to play a role in the formation of PSA [4, 8–10]. Patients with PSA can be either asymptomatic or symptomatic [7]. The diagnosis is based on color Doppler ultrasound examination [11–15]. Although PSAs can spontaneously thrombose, untreated cases may lead to rupture or get infected; therefore, most patients undergo some kind of treatment, such as the replacement of the pressure bandage, ultrasound-guided compression (UGC), ultrasound-guided thrombin injection (UGTI), and/or open surgical repair [4–7, 11–15].

Since most publications are limited to case series and studies with a small sample size, we aimed to identify risk factors for PSA development and determine their incidence in a large patient population in a high-volume, multidisciplinary, tertiary center.

## Patients and methods

### Study characteristics

Electronic medical records of 30,196 patients who underwent any type of radiological or cardiac endovascular procedure, including electrophysiological interventions, that required arterial puncture between January 2016 and May 2020 at the Heart and Vascular Center of Semmelweis University were retrospectively reviewed for PSA development. In addition, PSAs resulting from procedures where arterial puncture was unintended were also collected. Institutional review board approval was granted (Approval No: 176/2020). Due to the retrospective nature of the study, no informed consent for analysis of data was obtained from patients. All data were fully anonymized before we accessed them.

### Endovascular procedure characteristics

Vascular access was obtained using the Seldinger technique, with or without ultrasound guidance, with a 21-gauge needle in the case of radial, ulnar, and brachial arteries and with an 18-gauge needle in the case of femoral arteries. Endovascular and electrophysiological procedures, which were carried out in a standard manner, were performed by vascular interventional radiologists, invasive cardiologists, or electrophysiologists. At each therapeutic intervention, the patient received at least 5,000 IU heparin intra-arterially, and some cardiology procedures also included administration of weight-based heparin, eptifibatide, prasugrel, or alteplase. If the patient was not already on single (acetylsalicylic acid [ASA], 100 mg/day orally or clopidogrel, 75 mg/day orally) or dual antiplatelet therapy, an oral or intravenous loading dose was given immediately following the procedure (ASA, 250–500 mg and/or clopidogrel, 300–600 mg).

### Postprocedural access site management

In our center, the management of the access site after the procedures was as follows: in the case of radial and ulnar arteries, a compression assist device (TR Band; SCW Medicath Ltd., Shenzhen, China or Radial Compression Hemostasis Device; Fervid Medical Technology Co., Guangdong, China) was applied over the puncture site for 2–4 hours, while in the case of brachial artery, manual compression followed by pressure bandage placement for 4–6 hours was the standard of care. Based on the preference of the performing physician, size of the sheath used, and severity of vessel calcification at the access site, in patients with femoral artery puncture, hemostasis was achieved either by manual compression, followed by pressure bandage placement for 6–8 hours, or the use of a vascular closure device (VCD; Angio-Seal; Terumo Corp., Tokyo, Japan, Exoseal; Cordis Corp., Hialeah, FL, USA, or Perclose ProGlide; Abbott Laboratories, Chicago, IL, USA).

### Pseudoaneurysm color Doppler ultrasound examination

In the presence of symptoms (pulsatile swelling, tenderness/pain, skin changes, neuropathy, claudication/limb ischemia, hemodynamic instability) [7] and/or bruit at the site of the sheath removal, color Doppler ultrasound scanning was carried out by a registered vascular technician or radiologist. The diagnosis of PSA was established when a ‘yin-yang’ sign was seen in a cystic structure (sac) adjacent to the punctured artery and a communicating channel (neck) between the cystic structure and the feeding artery with a ‘to-and-fro’ waveform was demonstrated [11–15]. In each patient, the size of the PSA sac, the number of compartments in the sac, the width and length of the PSA neck, and the distance of the top of the PSA sac from the skin surface were assessed.

### Pseudoaneurysm treatment

Patients with asymptomatic PSA underwent treatment only if the PSA did not resolve and showed an increase in size within a week or if the patient became symptomatic [7]. Patients with symptomatic PSA were treated with replacement of the pressure bandage, UGC, UGTI, or open surgical repair [1, 4–7]. In patients whose coagulation status was normal, in whom PSA was detected within a week after the procedure, the PSA sac was superficial, simplex, and small (<20 mm), and the PSA neck was long and narrow, replacement of the pressure bandage or UGC was attempted [7, 16]. In other cases, UGTI or open surgical repair was carried out. Open surgical repair was mainly performed when the PSA caused local mass effect complications, was infected, had a short (<4 mm) and wide neck, or other minimally invasive techniques failed [16, 17]. UGC and UGTI were executed as described by Fellmeth et al. in 1991 and Cope and Zeit et al. in 1986 [18, 19]. In the case of UGTI, 500–1000 U/mL bovine thrombin (Tisseel tissue adhesive; Baxter Healthcare Corp., Largo, FL, USA) was injected into the PSA sac. Open surgical repair meant a simple suture of the arterial defect, patch plasty, or interposition grafting. The success of replacement of the pressure bandage, UGC, and UGTI was checked by color Doppler ultrasound examination after 24 hours. Thereafter, imaging was performed only for relevant complaints or symptoms.

### Data collection

The following data were collected from our medical record archiving system (MedSol; T-Systems Hungary Ltd., Budapest, Hungary): 1) patient demographics (date of birth, sex, weight, height, medical history, medication regimen); 2) preprocedural blood test results (white blood cell [WBC] count, red blood cell [RBC] count, hematocrit [HCT], hemoglobin [Hb], platelet count, prothrombin activity, international normalized ratio [INR], creatinine, glomerular filtration rate [GFR]); 3) endovascular procedure characteristics (type of the treatment, site and direction of the puncture [antegrade versus retrograde], size of the sheath used, sheath replacement [yes or no], time spent by the sheath in the artery, use of a VCD [yes or no]); and 4) PSA-related information (symptoms [yes or no], site of the PSA, size of the PSA sac and neck, number of the compartments in the sac, distance of the top of the PSA sac from the skin surface, type and outcome of the treatment).

Blood count parameters were measured using the Sysmex automated hematology analyzer (Sysmex Corp., Kobe, Japan), while coagulation parameters were determined by the STA Compact Max (Stago, Asnières-sur-Seine, France) fully automated benchtop analyzer.

### Statistical analyses

For statistical analysis, R version 4.0.0 (released on April 24, 2020) was used (R Core Team [2020]; R: A language and environment for statistical computing; R Foundation for Statistical Computing, Vienna, Austria). A control group of 134 patients was created to reveal predictors of PSA formation. Controls were randomly selected in a 1:1 fashion matched according to age, gender, and type of the procedure either from the study population (for patients with PSA in whom the arterial puncture was intended, N = 109) or from our medical record archiving system (for patients with PSA in whom the arterial puncture was unintended, N = 25). (Table 1) In the case of descriptive statistics, categorical data were presented as numbers (percentages), while continuous data were presented as means (± standard deviation [SD]) and medians (interquartile range [IQR]). In Table 1, P-values were based on the chi-squared test for categorical variables and the Welch’s t-test for continuous variables. Furthermore, the chi-squared test was also used to compare the prevalence of PSA among the treatment groups and to compare the prevalence of PSA in patients with and without VCD.

**Table 1:** Characteristics of the pseudoaneurysm and the control group.

Parameters	Patients with PSA (N = 134)	Control group (N = 134)	P-value
*Demographic parameters*			
Age (years)			1.000
Mean (SD)	69.5 (15.2)	69.5 (15.2)	
Median (IQR)	72.5 (15.8)	72.5 (15.8)	
Female sex, N (%)	72 (53.7)	72 (53.7)	1.000
*Atherosclerotic risk factors*			
BMI >30 kg/m^2^, N (%)	28 (20.9)	25 (18.7)	0.759
Smoking, N (%)	76 (56.7)	77 (57.5)	>0.999
Hypertension, N (%)	123 (91.8)	115 (85.8)	0.175
Dyslipidemia, N (%)	51 (38.1)	45 (33.6)	0.446
Diabetes mellitus, N (%)	37 (27.6)	39 (29.1)	0.892
Chronic kidney disease, N (%)	37 (27.6)	40 (29.9)	0.787
*Preprocedural blood test parameters*			
White blood cells (G/L)			0.160
Mean (SD)	8.47 (3.45)	7.87 (3.55)	
Median (IQR)	8.05 (3.25)	7.39 (3.54)	
Red blood cells (T/L)			<0.001
Mean (SD)	4.13 (0.80)	4.59 (0.51)	
Median (IQR)	4.01 (1.12)	4.60 (0.65)	
Hematocrit (L/L)			<0.001
Mean (SD)	0.37 (0.06)	0.41 (0.05)	
Median (IQR)	0.37 (0.08)	0.42 (0.05)	
Hemoglobin (g/dL)			<0.001
Mean (SD)	122 (22.4)	138 (15.2)	
Median (IQR)	122 (32.0)	139 (19.5)	
Platelets (G/L)			0.546
Mean (SD)	227 (86.4)	233 (72.6)	
Median (IQR)	206 (99.0)	222 (90.3)	
Prothrombin activity (%)			<0.001
Mean (SD)	81.7 (18.7)	89.9 (14.3)	
Median (IQR)	83.0 (25.5)	92.0 (7.8)	
International normalized ratio (IU)			<0.001
Mean (SD)	1.19 (0.25)	1.10 (0.17)	
Median (IQR)	1.14 (0.24)	1.06 (0.08)	
Creatinine (μmol/L)			0.005
Mean (SD)	129.0 (144.0)	90.2 (59.9)	
Median (IQR)	89.0 (48.5)	77.5 (26.5)	
*Procedure types*			
Vascular radiological, N (%)	53 (39.6)	53 (39.6)	1.000
Coronary artery, N (%)	31 (23.1)	31 (23.1)	1.000
Non-coronary artery cardiac, N (%)	25 (18.7)	25 (18.7)	1.000
Others, N (%)	25 (18.7)	25 (18.7)	1.000
*Access site*			
Upper extremity, N (%)	34 (25.4)	30 (22.4)	0.667
*Sheath size*			
>8F, N (%)	9 (6.7)	11 (8.2)	0.816
*Closure device*, *N (%)*	37 (27.6)	14 (10.4)	<0.001

*BMI*, Body mass index; *IQR*, interquartile range; *PSA*, pseudoaneurysm; *SD*, standard deviation.

To determine which parameters had a significant effect on PSA formation and how strong the effect was a logistic regression model was used. Three final models were created to avoid the multicollinearity problem arising from the high correlation between RBC count, HCT, and Hb values. The models contained WBC, RBC (or HCT or Hb), and platelet count; INR and creatinine values; body mass index (BMI) >30 kg/m^2^; atherosclerotic risk factors/comorbidities (smoking, hypertension, dyslipidemia, diabetes mellitus, and chronic kidney disease); puncture site; and sheath size. For statistical analysis, ANOVA and likelihood-ratio tests were calculated (using package car [version 3.0.8] with the function Anova). A variable effect was handled significant if the P-value of the likelihood-ratio test was <0.05. Effect sizes were expressed as odds ratios (ORs; with PSA versus without PSA).

## Results

**Fig 1:**
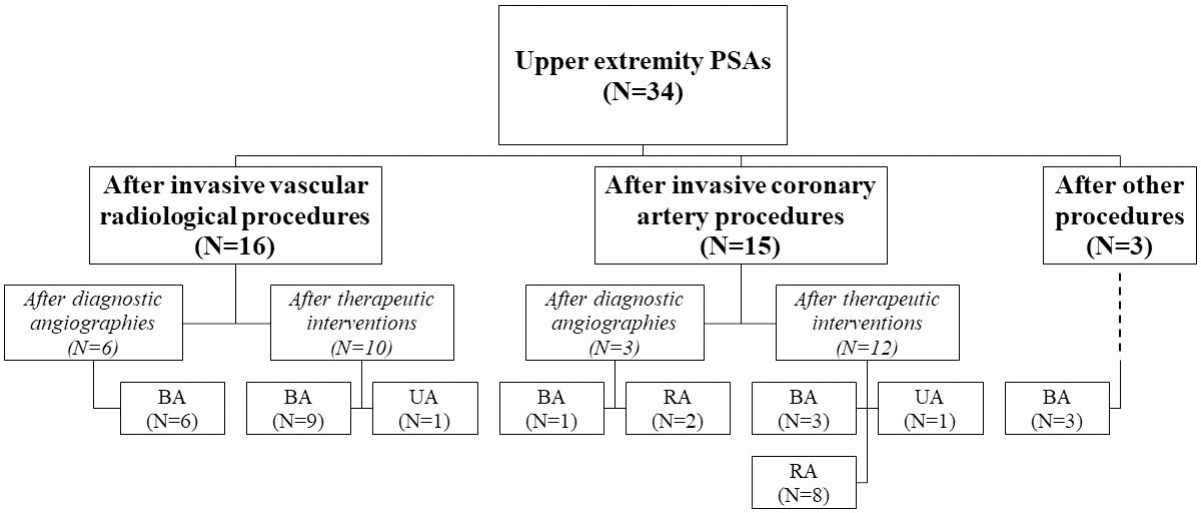
Upper extremity pseudoaneurysms. *BA*, Brachial artery; *PSA*, pseudoaneurysm; *RA*, radial artery; *UA*, ulnar artery.

**Fig 2:**
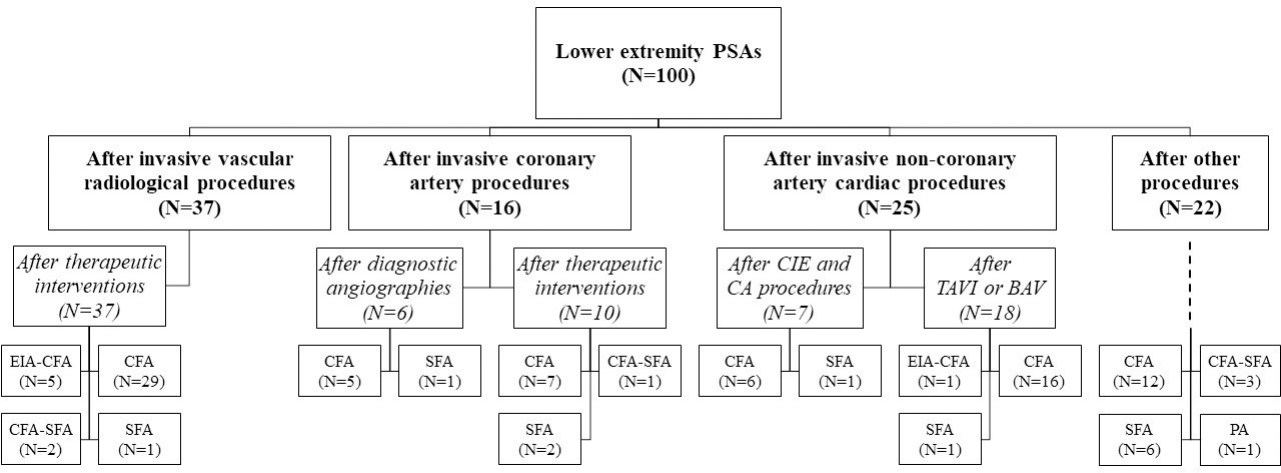
Lower extremity pseudoaneurysms. *BAV*, Balloon aortic valvuloplasty; *CA*, catheter ablation; *CFA*, common femoral artery; *CIE*, clinical intracardiac electrophysiological; *EIA*, external iliac artery; *PA*, popliteal artery; *PSA*, pseudoaneurysm; *SFA*, superficial femoral artery; *TAVI*, transcatheter aortic valve implantation.

During the examined period, 134 PSAs were identified in 134 patients. Fifty-three PSAs developed after invasive vascular radiological procedures (53/6555 [0.8%]), 31 after invasive coronary artery procedures (31/18038 [0.2%]), and 25 after invasive non-coronary artery cardiac procedures (25/5603 [0.4%]). In addition, 25 PSA cases were due to procedures in which the arterial puncture was unintended. The incidence of PSA was significantly different between the vascular radiological and the coronary artery group (P<0.001), between the vascular radiological and the non-coronary artery cardiac group (P = 0.038), and between the coronary artery and the non-coronary artery cardiac group (P = 0.001). Thirty-four PSAs (25.4%) were localized to the upper extremity arteries, while 100 (74.6%) arose from the lower extremity arteries. (Figs 1 and 2)

The mean age of the 134 patients (women, N = 72; men, N = 62) was 69.5 (±15.2) years. Twenty-eight of them (20.9%) were obese (BMI >30 kg/m^2^), 76 (56.7%) were smokers, 123 (91.8%) had hypertension, 51 (38.1%) had dyslipidemia, 37 (27.6%) had diabetes mellitus, and 37 (27.6%) had chronic kidney disease. (Table 1) The preprocedural blood test results of the patients can be found in Table 1, while their medication regimen is listed in Table 2.

**Table 2:** Medication regimen of patients with a pseudoaneurysm.

Medications	Patients with an upper extremity PSA (N = 34)	Patients with a lower extremity PSA (N = 100)
ASA monotherapy, N (%)	1 (2.9)	14 (14)
Clopidogrel monotherapy, N (%)	2 (5.9)	6 (6)
Dual antiplatelet therapy, N (%)	11 (32.4)	24 (24)
Cilostazol therapy, N (%)	1 (2.9)	0 (0)
Dual antiplatelet therapy + cilostazol therapy, N (%)	0 (0)	2 (2)
Conventional anticoagulant therapy, N (%)	5 (14.7)	15 (15)
Mono antiplatelet therapy + conventional anticoagulant therapy, N (%)	7 (20.6)	13 (13)
Dual antiplatelet therapy + conventional anticoagulant therapy, N (%)	5 (14.7)	1 (1)
NOAC therapy, N (%)	0 (0)	16 (16)
Mono antiplatelet therapy + NOAC therapy, N (%)	0 (0)	8 (8)
Dual antiplatelet therapy + NOAC therapy, N (%)	1 (2.9)	3 (3)

*ASA*, Acetylsalicylic acid; *NOAC*, novel oral anticoagulant; *PSA*, pseudoaneurysm.

### Upper extremity PSAs

Sixteen (47.1%) of 34 upper extremity PSAs were observed after invasive vascular radiological procedures, 15 (44.1%) after invasive coronary artery procedures, and three (8.8%) were due to procedures in which the arterial puncture was unintended. These procedures included the placement of a peripheral intravenous catheter (N = 2) and cannulation of a fistula for dialysis (N = 1). In four patients (4/31 [12.9%]), PSA occurred after an antegrade puncture. The site of the antegrade puncture was the brachial artery and all four patients had hemodialysis fistula angioplasty.

Localization and sheath-related parameters are shown in Fig 1 and Table 3. VCD was not used in any of the patients.

**Table 3:** Localization and sheath-related parameters of patients with an upper extremity pseudoaneurysm.

Localization, sheath-related parameters	Patients with an upper extremity PSA (N = 34)
*Localization*	
Brachial artery, N (%)	22 (64.7)^a^
Radial artery, N (%)	10 (29.4)^b^
Ulnar artery, N (%)	2 (5.9)^c^
*Sheath size* ^d^	
4F, N (%)	4/31 (12.9)
5F, N (%)	7/31 (22.6)
6F, N (%)	17/31 (54.8)
7F, N (%)	2/31 (6.5)
8F, N (%)	1/31 (3.2)
*Sheath replacement*, N (%)	11/31 (35.5)
*Time spent by the sheath in the artery* (minutes), mean (±SD)	31.3 (24.4)

*PSA*, Pseudoaneurysm; *SD*, standard deviation.

^a^ 22/1897 (1.2%)—all brachial artery punctures.

^b^ 10/20478 (0.05%)—all radial artery punctures.

^c^ 2/1818 (0.1%)—all ulnar artery punctures.

^d^ The largest sheath size used was counted for each patient.

The mean diameter of the PSA sac was 22.4 (±14.5) mm, while the mean length of the PSA sac was 13.1 (±8.8) mm. The mean width of the PSA neck was 2.6 (±0.8) mm, while the mean length of the PSA neck was 7.3 (±7.2) mm. PSA with more than one compartment was noted in two patients (5.9%). The mean distance of the top of the PSA sac from the skin surface was 6.2 (±3.9) mm.

The indication for PSA treatment was a non-disappearance of the sac in two cases (5.9%), an increase in the size of the sac in four (11.8%), and a symptom in 28 (82.4%). Eleven PSAs (32.4%) were treated with replacement of the pressure bandage, four (11.8%) with UGC, 13 (38.2%) with UGTI, and six (17.6%) with open surgical repair (simple suture of the arterial defect, N = 6). The first elimination attempt of the PSA was not successful in three patients (8.8%). In two cases, it was the replacement of the pressure bandage, while in one, UGTI failed. The final solution for those in whom the replacement of the pressure bandage did not lead to the disappearance of PSA was UGTI, while the third patient underwent interposition grafting. The primary success rate of the replacement of the pressure bandage was 81.8%, UGC was 100%, UGTI was 92.3%, and open surgical repair was 100%. No complications were observed during PSA treatments.

### Lower extremity PSAs

Thirty-seven (37%) of 100 lower extremity PSAs were observed after invasive vascular radiological procedures, 16 (16%) after invasive coronary artery procedures, 25 (25%) after invasive non-coronary artery cardiac procedures, and 22 (22%) were due to procedures in which the arterial puncture was unintended. These procedures included certain types of electrophysiological treatments for arrhythmias (N = 20), cannulation of a deep vein for a catheter-directed thrombolysis (N = 1), and placement of a central venous catheter for therapeutic plasma exchange (N = 1). The prevalence of femoral artery PSA was 0.4% (99/22202) in the whole patient group, while it was 2.9% (18/630) in a subgroup of patients with balloon aortic valvuloplasty (BAV) or transcatheter aortic valve implantation (TAVI). In TAVI, both femoral arteries were punctured; therefore, the PSA prevalence for the punctured artery was 1.2% (14/1126) in the case of TAVI. The femoral artery PSA prevalence for the punctured artery was 0.4% (81/21009) for the other procedures. In 11 patients (11/78 [14.1%]), PSA occurred after an antegrade puncture. The intended site of the antegrade puncture was the common femoral artery in all patients.

Localization, sheath- and VCD-related parameters can be seen in Fig 2 and Table 4. The prevalence of PSA for the punctured artery with and without VCD use was 37/3555 (1%) and 97/27204 (0.4%), respectively (OR, 2.94; 95% confidence interval, 1.95–4.34; P<0.001).

**Table 4:** Localization, sheath- and vascular closure device-related parameters of patients with a lower extremity pseudoaneurysm.

Localization, sheath- and VCD-related parameters	Patients with a lower extremity PSA (N = 100)
*Localization*	
Transition between the EIA and the CFA, N (%)	6 (6)
CFA, N (%)	75 (75)
Transition between the CFA and the SFA, N (%)	6 (6)
SFA, N (%)	12 (12)
Popliteal artery, N (%)	1 (1)
*Sheath size* ^a^	
4F, N (%)	1/78 (1.3)
5F, N (%)	11/78 (14.1)
6F, N (%)	55/78 (70.5)
8F, N (%)	2/78 (2.6)
9F, N (%)	2/78 (2.6)
12F, N (%)	1/78 (1.3)
14F, N (%)	1/78 (1.3)
16F, N (%)	5/78 (6.4)
*Sheath replacement*, N (%)	19/78 (24.4)
*Time spent by the sheath in the artery* (minutes), mean (±SD)	55.4 (34.7)
*PSA development despite the use of a VCD*, N (%)	37 (37)
*VCD type*	
Angio-Seal, N (%)	30/37 (81.1)^b^
Perclose ProGlide, N (%)	6/37 (16.2)^c^
Exoseal, N (%)	1/37 (2.7)^d^

*CFA*, Common femoral artery; *EIA*, external iliac artery; *PSA*, pseudoaneurysm; *SD*, standard deviation; *SFA*, superficial femoral artery; *VCD*, vascular closure device.

^a^ The largest sheath size used was counted for each patient.

^b^ 30/2894 (1%)—all Angio-Seal cases.

^c^ 6/563 (1.1%)—all Perclose ProGlide cases.

^d^ 1/98 (1%)—all Exoseal cases.

The mean diameter of the PSA sac was 27 (±16.1) mm, while the mean length of the PSA sac was 19 (±11.4) mm. The mean width of the PSA neck was 3.3 (±1.3) mm, while the mean length of the PSA neck was 10.8 (±7.1) mm. PSA with more than one compartment was noted in 30 patients (30%). The mean distance of the top of the PSA sac from the skin surface was 13.2 (±7.8) mm.

The indication for PSA treatment was a non-disappearance of the sac in six cases (6%), an increase in the size of the sac in 10 (10%), and a symptom in 84 (84%). Fourteen PSAs (14%) were treated with replacement of the pressure bandage, one (1%) with UGC, 73 (73%) with UGTI, and 12 (12%) with open surgical repair (simple suture of the arterial defect, N = 8; patch plasty, N = 2; interposition grafting, N = 2). The first elimination attempt of the PSA was not successful in 16 patients (16%). In six cases, it was the replacement of the pressure bandage, while in 10, UGTI failed. The final solution for those whose PSA did not disappear after the first elimination attempt was either UGTI (N = 3) or simple suture of the arterial defect (N = 13). The primary success rate of the replacement of the pressure bandage was 57.1%, UGC was 100%, UGTI was 86.3%, and open surgical repair was 100%.

### Comparison of patients with and without PSA

Characteristics of the PSA and the control group can be seen in Table 1.

**Fig 3:**
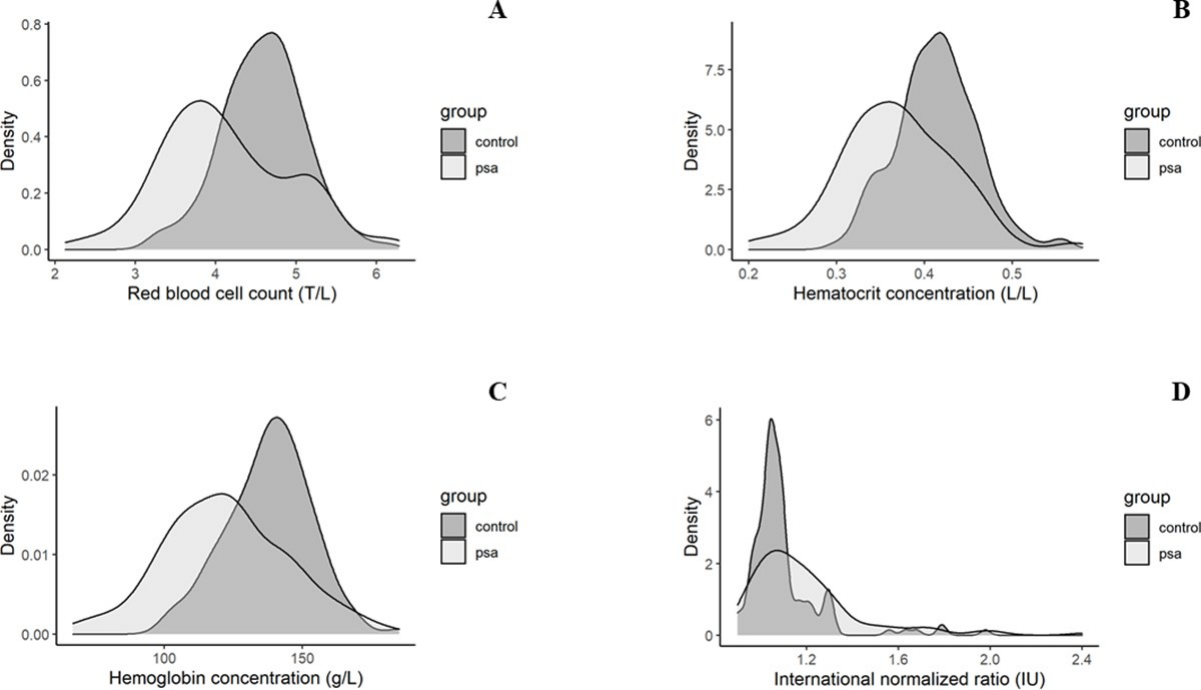
The single effect of red blood cells (A), hematocrit (B), hemoglobin (C), and international normalized ratio (D) on pseudoaneurysm development. Kernel density plots of distributions.

The effect of RBC count (OR, 0.33; at average INR value), HCT value (OR, 0.87; at average INR value), Hb value (OR, 0.96; at average INR value), INR (OR, 12.97; at average RBC count), RBC count—INR interaction (OR, 22.28) (or HCT value—INR interaction [OR, 1.35] or Hb value—INR interaction [OR, 1.08]), and RBC count—VCD use interaction (OR, 3.27) (or HCT value—VCD use interaction [OR, 1.17] or Hb value—VCD use interaction [OR, 1.05]) on PSA formation was significant. (Table 5) The single effect of RBC count, HCT, Hb, and INR values on PSA development is shown in Fig 3. Neither other laboratory parameters, nor atherosclerotic risk factors/comorbidities, site of the puncture, and size of the sheath used showed association with the presence of PSA.

**Table 5:** Predictors of pseudoaneurysm formation.

Parameters	OR	95% CI	P-value
BMI >30 kg/m^2^	1.19	0.56–2.53	0.626
Smoking	1.06	0.60–1.88	0.835
Hypertension	1.94	0.77–4.93	0.153
Dyslipidemia	1.37	0.78–2.44	0.276
Diabetes mellitus	0.73	0.39–1.38	0.332
Chronic kidney disease	0.76	0.41–1.44	0.403
WBC count	1.06	0.97–1.16	0.190
RBC count	0.33	0.21–0.52	<0.001
HCT value	0.87	0.82–0.91	<0.001
Hb value	0.96	0.94–0.97	<0.001
Platelet count	1.00	0.10–1.00	0.857
INR	12.97	2.58–65.30	<0.001
Creatinine level	1.00	0.10–1.01	0.059
Upper extremity access	1.12	0.58–2.16	0.727
>8F sheath	1.01	0.35–2.95	0.985
Closer device	3.58	1.71–7.47	<0.001
RBC count—INR interaction	22.28	2.91–170.61	0.003
HCT value—INR interaction	1.35	1.07–1.71	0.014
Hb value—INR interaction	1.08	1.01–1.16	0.036
RBC count—closer device use interaction	3.27	1.09–9.80	0.036
HCT value—closer device use interaction	1.17	1.02–1.34	0.022
Hb value—closer device use interaction	1.05	1.01–1.09	0.012

*BMI*, Body mass index; *CI*, confidence interval; *Hb*, hemoglobin; *HCT*, hematocrit; *INR*, international normalized ratio; *OR*, odds ratio; *RBC*, red blood cell; *WBC*, white blood cell.

## Discussion

The incidence of PSA was significantly higher in vascular radiological than in coronary artery procedures, which can be explained by the higher percentage of therapeutic interventions (4180/6555 [63.8%] versus 7709/18038 [42.7%], respectively) and by the more common use of brachial and femoral access in the vascular radiological group (4181/6555 [63.8%] versus 1419/18038 [7.9%], respectively). In therapeutic interventions, compared to diagnostic angiographies, sheaths are generally larger in caliber, device changes are more frequent, and more time is required to perform them; all of which are known to predispose patients to PSA development [4–7]. The reason for the difference in PSA incidence between the coronary artery and non-coronary artery cardiac groups may be similar (therapeutic interventions, 7709/18038 [42.7%] versus 5603/5603 [100%]; brachial and femoral access, 1419/18038 [7.9%] versus 1430/5603 [25.5%]). The higher PSA incidence of vascular radiological procedures compared to non-coronary artery cardiac interventions may be due to the fact that the majority of vascular radiological procedures are indicated for atherosclerotic steno-occlusive disease, so the punctured arteries of this patient population may be more calcified and consequently more prone to PSA formation.

In the upper limb, the radial and brachial arteries are the most common puncture sites, but the ulnar artery is also increasingly used [8–10, 20, 21]. Radial and ulnar punctures have a lower major complication rate than brachial and femoral punctures [9, 10, 20–23]. Two complications related to radial and ulnar puncture, spasm and occlusion, should be highlighted [20–23], but they rarely cause clinical symptoms as the hand has a dual arterial blood supply. The incidence of radial and ulnar PSA is low, less than 0.1% [20–23]. Major complications associated with the brachial approach include local thrombus formation, PSA, and nerve compression by hematoma and/or PSA [8, 10]. In a study by Tamanaha et al. [8], the prevalence of PSA and nerve compression by PSA was significantly higher in brachial (1.1% and 2.2%, respectively) than in femoral access (0.4% and 0%, respectively). In another study comparing three puncture sites (radial, brachial, and femoral), the brachial puncture site had the highest risk of developing a large hematoma (0.7%, 4.4%, and 1.5%, respectively) or PSA (0%, 1.3%, and 0%, respectively) [10]. Our results were consistent with literature data, as the order of PSA incidence in the upper limb was as follows: 1) brachial artery (1.2%), 2) ulnar artery (0.1%), and 3) radial artery (0.05%).

The femoral approach is still widely used, especially when the procedure requires a large sheath (e.g., BAV, TAVI). Additionally, most lower extremity interventions are performed through the common femoral artery [24]. The incidence of femoral PSA is 0.05–2% after peripheral artery or coronary artery diagnostic catheterizations [25] and 2–6% after therapeutic interventions [7]. In patients with non-coronary artery cardiac interventions, such as intracardiac electrophysiological procedures or TAVI, the prevalence of femoral PSA was 0.3–0.9% [26, 27] and 1.6–5.9% [28, 29], respectively. Our study revealed a 0.4% incidence of femoral PSA, while it was 2.9% in a subgroup of patients who underwent BAV or TAVI.

VCDs are most commonly used to close femoral punctures. The use of VCD reduces the time to hemostasis, thus facilitating early patient mobilization, eliminating discomfort caused by prolonged bed rest, and shortening hospital stay length [30]. Although VCDs reduce the overall number of puncture-related complications, they slightly increase the risk of PSA formation [30–32]. The exact mechanism by which the use of VCD leads to the development of PSA is unknown. Presumably, it is not the VCD itself but the choice of the wrong type and size or its incorrect use that results in PSA.

Replacement of the pressure bandage is still often used with 23.2–98.1% efficacy for the therapy of PSAs [33–36]. In our study, the primary success rate of mechanical compression in the upper limb (81.8%) was higher than in the lower limb (57.1%), which may be because the punctured arteries in the upper limb were generally closer to the skin surface than in the lower limb. Over the years, the traditional open surgical repair of PSAs has been replaced by less invasive therapies, such as UGC and UGTI [15]. Other minimally invasive techniques, such as Angio-Seal or Perclose ProGlide, have only been tried on a few patients [37]. UGTI has a higher success rate (89–100%) than UGC (57–99%), especially if the PSA is large and/or if the patient is anticoagulated [38]. For UGTI, we showed a primary success rate of 92.3% for the upper limb and 86.3% for the lower limb, while for UGC, we observed a primary success rate of 100% for both the upper and lower limbs.

Several studies have reported an association between anemia and an increased number of perioperative complications in patients undergoing either cardiac or non-cardiac surgery [39–41]. In this study, a low RBC count and low HCT and Hb levels were found to be predictive factors for PSA formation. RBCs have an important rheological effect, interacting with endothelial cells, platelets, and fibrin(ogen) both directly and indirectly. Their aggregation and deformability cause laminar shearing with platelet margination [42, 43]. HCT, like RBCs, has been shown to promote the transport of platelets to the site of vessel wall injury, thereby enhancing their interaction with the endothelium [42, 44]. In anemia, the reduced effect of RBCs and HCT on platelets may increase the risk of post-puncture PSA development. The relationship between intracellular Hb and PSA occurrence is unclear and is probably related to anemia.

INR level had a significant effect on PSA development. The correlation of higher INR values with bleeding complications is well known [45]. A meta-analysis by Popma et al. [46] demonstrated that patients undergoing coronary artery intervention with an INR >3 had a three-fold increased risk of bleeding events compared to those with an INR ≤3. The role of high INR values in the formation of PSA [4, 47] and the need for reintervention after UGTI [33] has already been described. High INR values presumably lead to PSA development through the delayed coagulation cascade.

Our study had two primary limitations: 1) it was a single-center, retrospective study, and 2) the patient population was heterogeneous due to the large number of cases.

In conclusion, the incidence of PSA was highest after radiological procedures. Patients in whom the puncture site is closed with a VCD require increased observation. Preprocedural laboratory findings are useful for the identification of patients at high risk of PSA formation.

## Supporting information

S1 Table Pseudoaneurysm and control group data. (XLS)
